# A dual-branch deep learning model based on fNIRS for assessing 3D visual fatigue

**DOI:** 10.3389/fnins.2025.1589152

**Published:** 2025-06-05

**Authors:** Yan Wu, TianQi Mu, SongNan Qu, XiuJun Li, Qi Li

**Affiliations:** ^1^School of Computer Science and Technology, Changchun University of Science and Technology, Changchun, China; ^2^Jilin Provincial International Joint Research Center of Brain Informatics and Intelligence Science, Changchun, China; ^3^Zhongshan Institute of Changchun University of Science and Technology, Zhongshan, China

**Keywords:** 3D visual fatigue, fNIRS (functional near-infrared spectroscopy), deep learning, spatiotemporal features, neuroimaging analysis

## Abstract

**Introduction:**

Extended viewing of 3D content can induce fatigue symptoms. Thus, fatigue assessment is crucial for enhancing the user experience and optimizing the performance of stereoscopic 3D technology. Functional near-infrared spectroscopy (fNIRS) has emerged as a promising tool for evaluating 3D visual fatigue by capturing hemodynamic responses within the cerebral region. However, traditional fNIRS-based methods rely on manual feature extraction and analysis, limiting their effectiveness. To address these limitations, a deep learning model based on fNIRS was constructed for the first time to evaluate 3D visual fatigue, enabling end-to-end automated feature extraction and classification.

**Methods:**

Twenty normal subjects participated in this study (mean age: 24.6 ± 0.88 years; range: 23–26 years; 13 males). This paper proposed an fNIRS-based experimental paradigm that acquires data under both comfort and fatigue conditions. Given the time-series nature of fNIRS data and the variability of fatigue responses across different brain regions, a dual-branch convolutional network was constructed to separately extract temporal and spatial features. A transformer was integrated into the convolutional network to enhance long-range feature extraction. Furthermore, to adaptively select fNIRS hemodynamic features, a channel attention mechanism was integrated to provide a weighted representation of multiple features.

**Results:**

The constructed model achieved an average accuracy of 93.12% within subjects and 84.65% across subjects, demonstrating its superior performance compared to traditional machine learning models and deep learning models.

**Discussion:**

This study successfully constructed a novel deep learning framework for the automatic evaluation of 3D visual fatigue using fNIRS data. The proposed model addresses the limitations of traditional methods by enabling end-to-end automated feature extraction and classification, eliminating the need for manual intervention. The integration of a transformer module and channel attention mechanism significantly enhanced the model’s ability to capture long-range dependencies and adaptively weight hemodynamic features, respectively. The high classification accuracy achieved within and across subjects highlights the model’s effectiveness and generalizability. This framework not only advances the field of fNIRS-based fatigue assessment but also provides a valuable tool for improving user experience in stereoscopic 3D applications. Future work could explore the model’s applicability to other types of fatigue assessment and further optimize its performance for real-world scenarios.

## 1 Introduction

Three-dimensional (3D) display technology has garnered widespread attention for its ability to enhance realism. However, prolonged viewing of 3D content, as compared to traditional 2D, often results in various symptoms of visual fatigue, such as headaches and eye pain. In some cases, it may even cause irreversible health damage. These issues greatly slow down the progress of stereoscopic display technologies (Karimi et al., 2021; Liu et al., 2021). Therefore, the assessment of stereoscopic visual fatigue is an important research area.

Functional near-infrared spectroscopy (fNIRS) is a non-invasive imaging method that uses near-infrared light to monitor hemodynamic responses in the cerebral cortex (Jobsis, 1977; Acuña et al., 2024). By measuring changes in oxyhaemoglobin (HbO), deoxyhaemoglobin (HbR), and total hemoglobin (HbT), fNIRS provides detailed insights into localized brain activity related to blood oxygenation and hemodynamic responses. Compared to other techniques, such as EEG and fMRI, fNIRS balances both temporal and spatial resolution. This combination makes fNIRS a preferred approach for studying brain activation during different cognitive and perceptual tasks, including tasks involving stereoscopic visual. Additionally, the convenience and resistance to interference of fNIRS further enhance its suitability. Ward et al. employed (fNIRS to explore the relationship between the parietal cortex and stereoscopic visual perception). They evaluated the efficacy of stereoscopic vision by analyzing the changes in the levels of oxyhemoglobin (HbO) and deoxyhemoglobin (HbR; Ward et al., 2016). Seraglia et al. discovered that when participants viewed identical scenes via virtual reality technology, the hemodynamic responses were more pronounced in comparison to when they viewed those scenes in the real world (Seraglia et al., 2011; Zhou and Wang, 2024). Shi et al. employed subjective questionnaires in combination with statistical characteristics including peak amplitude (PA) and peak time (PT) for the purpose of assessing the effect exerted by color saturation on visual fatigue. The findings revealed that the peak amplitude (PA) of the HbO signal demonstrated a significant divergence between the visual comfort and visual fatigue conditions. Moreover, these differences were far more conspicuous than those detected in subjective evaluations and other statistical characteristics of the signal (Shi et al., 2022). Cai et al. utilized fNIRS to investigate the correlation between visual fatigue and cortical neural activity. By integrating statistical parameter analysis with the observation of the signal variation curve in response to visual stimuli, they discerned significant differences between the signals associated with visual comfort and those associated with visual fatigue (Cai et al., 2017). Hans et al. explored the mechanisms by which vergence-accommodation conflict gives rise to stereoscopic visual fatigue and impacts the HbO signals within the prefrontal cortex. Statistical analysis results indicated that larger vergence amplitudes were associated with more severe fatigue and more pronounced changes in HbO concentration (Howe et al., 2013). Yao et al. pointed out that subsequent to watching 3D movies, the frontal lobe undergoes robust activation, accompanied by a notable increase in HbO levels. Through the selection of pertinent signal features and the application of machine - learning techniques, it becomes possible to accurately classify the signals prior to and subsequent to viewing 3D content (Yao et al., 2022). Despite the remarkable advancements in this field, the fNIRS research focusing on stereoscopic visual fatigue still relies on manual feature selection and analysis of changes within the Region of Interest (ROI) to evaluate its impact on brain activity. Manual feature extraction, which relies on prior knowledge, is time-consuming and prone to human error. These limitations hinder its application in large-scale data and complex tasks. The time-series nature of fNIRS signals contains latent features which are not easily understood by artificial stereoscopic visual fatigue still relies on manual feature selection and analysis of changes within the Region of Interest (ROI) to evaluate its impact on brain activity.

In recent years, deep learning techniques have attained remarkable feats across a diverse range of domains, such as computer visual, speech recognition, and time series classification (TSC; Zhu et al., 2022; Xin et al., 2023; Shahabaz and Sarkar, 2024). Unlike traditional models that require manual feature extraction, deep learning models can directly extract feature representations from raw data, enabling end-to-end learning. Deep learning optimizes both feature extraction and classification, overcoming the limitations of traditional manual methods and demonstrating strong generalization across various tasks and datasets (Lawhern et al., 2018; Zhang et al., 2020; Sedik et al., 2023). Considering the time-series nature of fNIRS signals, Convolutional Neural Networks (CNNs) have significant advantages. By capturing local temporal dependencies and identifying relevant features within adjacent time intervals, CNNs have become the most common model for time series classification tasks based on fNIRS (Dewen et al., 2021; Ma et al., 2021). However, CNN’s capture of local temporal dependencies may overlook long-term temporal dependencies related to fatigue (such as fatigue accumulation effects), while LSTM can model long-range time series, its sequence to sequence architecture cannot effectively integrate the multi-channel spatial information of fNIRS. Furthermore, upon continuous stereoscopic visual stimulation, fatigue characteristics gradually build up in both the temporal and spatial dimensions. In terms of temporal dynamics, fatigue development requires time-dependent modeling, but the receptive field of CNN is limited by the size of the convolution kernel and the depth of the network. In the spatial domain, different brain regions have different response patterns to visual stimuli, requiring independent optimization of spatial feature extraction. However, a single branch of CNN cannot separate specialized processing of spatiotemporal features. Therefore, a dual branch structure is adopted to capture temporal and spatial features separately. Specifically, we engineered a Dual - Branch Convolutional Neural Network (DBCNN) feature extraction module. This module is intended to bolster the proficiency of CNNs in discerning and harnessing the characteristic features embedded in fNIRS data related to stereoscopic visual fatigue.

Given that research on visual fatigue necessitates sustained stimuli to promote the accumulation of fatigue, CNNs mainly focus on extracting local features from the data. This characteristic restricts their capacity to process global information effectively. In contrast, Transformer, a deep model based on self- attention mechanism, exhibit significant advantages in capturing global context (Chen et al., 2023; Wang H. et al., 2023; Lin et al., 2024). This capability is particularly advantageous for analyzing stereoscopic visual fatigue data, which requires extensive accumulation over prolonged periods. Given the prowess of CNNs in local feature extraction and the capabilities of Transformers in global context modeling, integrating these two modules enables the exploitation of the respective advantages of CNNs and Transformers. As a result, remarkable classification outcomes can be achieved (Huang et al., 2022; Song et al., 2023; Zhang et al., 2023).

When choosing fNIRS hemodynamic signals, it is crucial to recognize that diverse hemodynamic features carry different levels of significance in the evaluation of stereoscopic visual fatigue. Existing studies that are based on fNIRS typically either choose only a single feature or utilize all available features for analysis (Dewen et al., 2021; Ma et al., 2023). Inspired by the image domain (27), we integrated the hemodynamic feature channel attention mechanism into the DBCNN module. Through the application of this attention mechanism, the module can autonomously optimize the input weights of various hemodynamic feature signals. We implemented this strategy to evaluate stereoscopic visual fatigue, thus improving the efficient use of fNIRS feature data and driving progress in the field (Wang, Y. Q. et al., 2023).

Therefore, a model integrating a dual-branch CNN, the hemodynamic feature channel attention mechanism, and Transformer is constructed for extracting features and classifying visual fatigue and comfort conditions. The primary contributions of this paper are summarized as follows:

1. For the first time, deep learning technology has been comprehensively integrated into the assessment of stereoscopic visual fatigue through fNIRS spectroscopy imaging. The proposed model seamlessly combines the DBCNN, a channel attention mechanism, and Transformer modules. By capitalizing on their individual capabilities in local and global feature extraction, this innovative approach remarkably improves the accuracy and efficiency of the stereoscopic visual fatigue assessment.
2. For the first time, the channel attention mechanism was introduced into the feature channels of fNIRS. This novel approach allows for more effective utilization of fNIRS hemodynamic feature data, thereby further enhancing the accuracy of the deep - learning model.
3. An fNIRS-based stereoscopic visual fatigue stimulation paradigm was developed, and signals corresponding to both comfort and fatigue states were collected.

## 2 Methods

### 2.1 Participants

Twenty participants (13 males and 7 females, aged 23–26 years; mean age 25 ± 0.88 years) were recruited from Changchun University of Science and Technology. All participants demonstrated normal or corrected-to-normal visual acuity (Snellen equivalent ≥20/25) to ensure full engagement in stereoscopic tasks. Prior to the experiment, eligibility was confirmed through preliminary screening tests, including visual function assessments and task familiarity evaluations. To minimize experimental variability, participants were instructed to refrain from strenuous activities and maintain adequate rest for 24 h before the sessions. Standardized protocols were implemented throughout the study to ensure consistent preparation and data collection.

During the experimental sessions, participants were seated in a height-adjustable chair positioned 285 cm from a stereoscopic display (ASUS VG278HR; screen dimensions: 95 cm height × 170 cm width). To standardize viewing conditions, a chin rest (HeadSpot®, UHCOTech) was utilized to minimize head motion artifacts and maintain a fixed distance of 60 cm between the nasion (bridge of the nose) and the display center. Participants’ viewing distance was three times the TV height (95 cm; Shin et al., 2021). This configuration ensured stable fNIRS signal acquisition by eliminating motion-induced optical path fluctuations while optimizing stereoscopic stimulus presentation accuracy.

### 2.2 General procedure

#### 2.2.1 Stimuli

In this experiment, we utilized static stereograms as the stimulus. Each stereogram consisted of two images exhibiting horizontal disparity, symmetrically shifted to the left and right relative to the background in order to create a binocular disparity of *α* + *β*. These images were presented separately to the left and right eyes, thereby eliciting a depth perception characteristic of stereoscopic vision. The stereograms were created and displayed using Unity 3D software. To simulate human binocular vision, two virtual cameras (left/right eye) were positioned in the 3D scene with an inter-pupillary distance (IPD) of 65 mm, consistent with anthropometric standards for adult populations [ISO 15099:2018]. The stereoscopic stimulus consisted of a single achromatic cube (RGB: [0, 255, 0]; luminance: 120 cd/m^2^; edge length: 2.3° visual angle) centered on a neutral gray background (RGB: [128, 128, 128]; luminance: 50 cd/m^2^), designed to isolate disparity cues while controlling for chromatic and contextual confounders.

**Figure 1:**
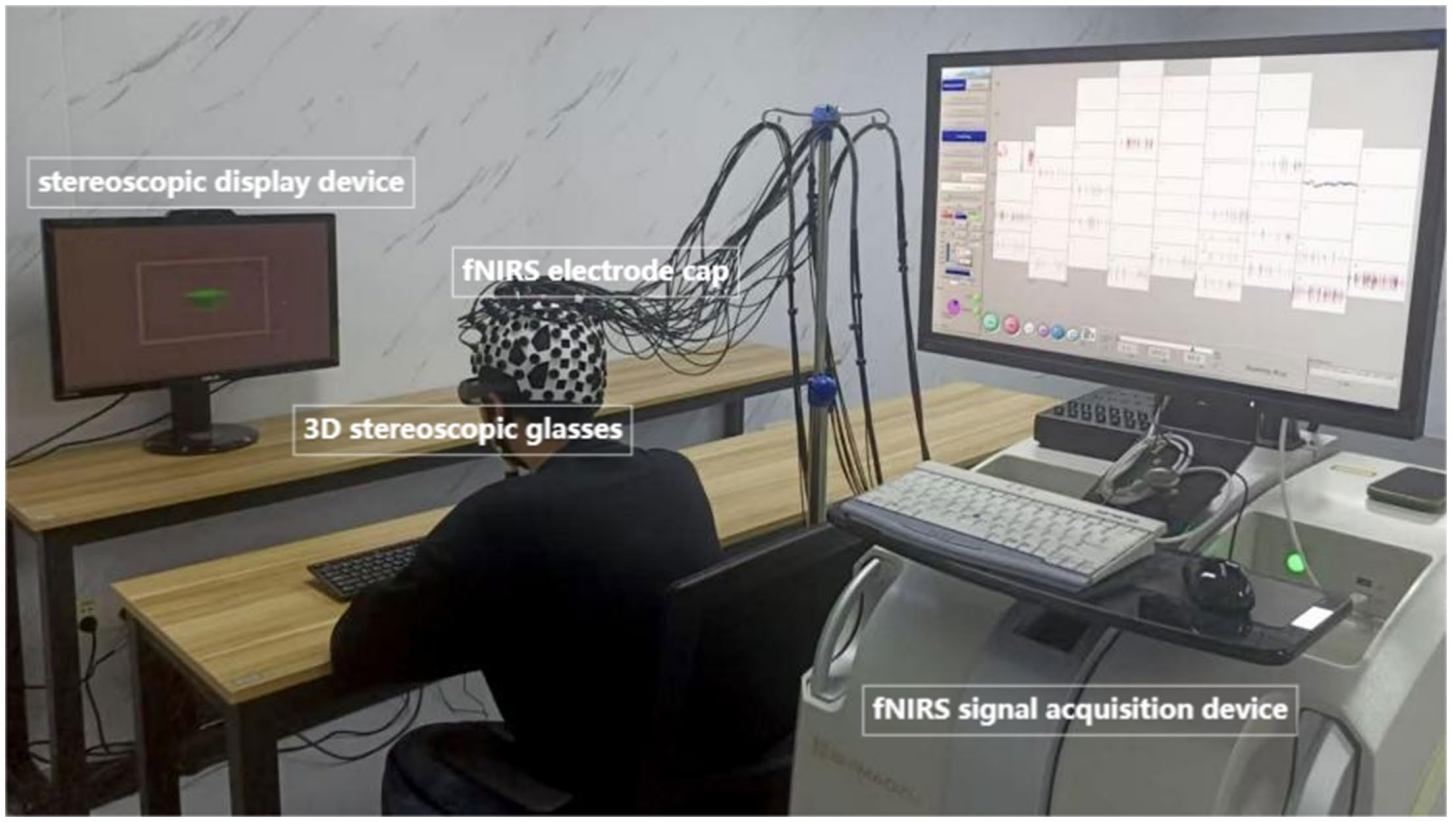
Experimental environment.

Previous research has relied on subjective measurements from questionnaire surveys to assess visual fatigue induced by vergence-accommodation conflicts (Kim et al., 2014; Yangyi et al., 2022; Watanabe et al., 2024). Although subjective assessments provide direct insights into participants’ perceptual experiences, they are susceptible to inter-individual variability in response bias and fatigue tolerance thresholds. To address these limitations, recent research has shifted toward objective physiological metrics, including pupillary dynamics, blink rate analysis, and ocular accommodation responses, which offer higher reliability and precision in quantifying VAC-induced visual fatigue. Studies have also indicated that static stereograms with larger disparities tend to induce more visual fatigue than those with smaller disparities (Zheng et al., 2024). Building on these findings, we designed the experimental stimuli to systematically manipulate binocular disparity across two conditions: visual comfort (VC) and visual fatigue (VF). For the VC condition, six stereograms with minimal disparity values (±0.1°, ±0.2°, ±0.3°) were selected, ensuring low vergence-accommodation conflict (VAC) demands. Conversely, for the VF condition, six stereograms with elevated disparity values (±1.0°, ±0.9°, ±0.8°) were employed, designed to induce measurable visual fatigue based on prior evidence [34]. This resulted in a total of 12 distinct disparity levels, each presented in randomized order to minimize habituation effects. To objectively quantify neural responses, fNIRS was utilized to record hemodynamic changes in the prefrontal cortex (PFC) and visual association areas during stimulus presentation. The experimental setup, including stimulus display parameters and fNIRS probe placement, is illustrated in Figure 1.

#### 2.2.2 fNIRS data recording

**Figure 2:**
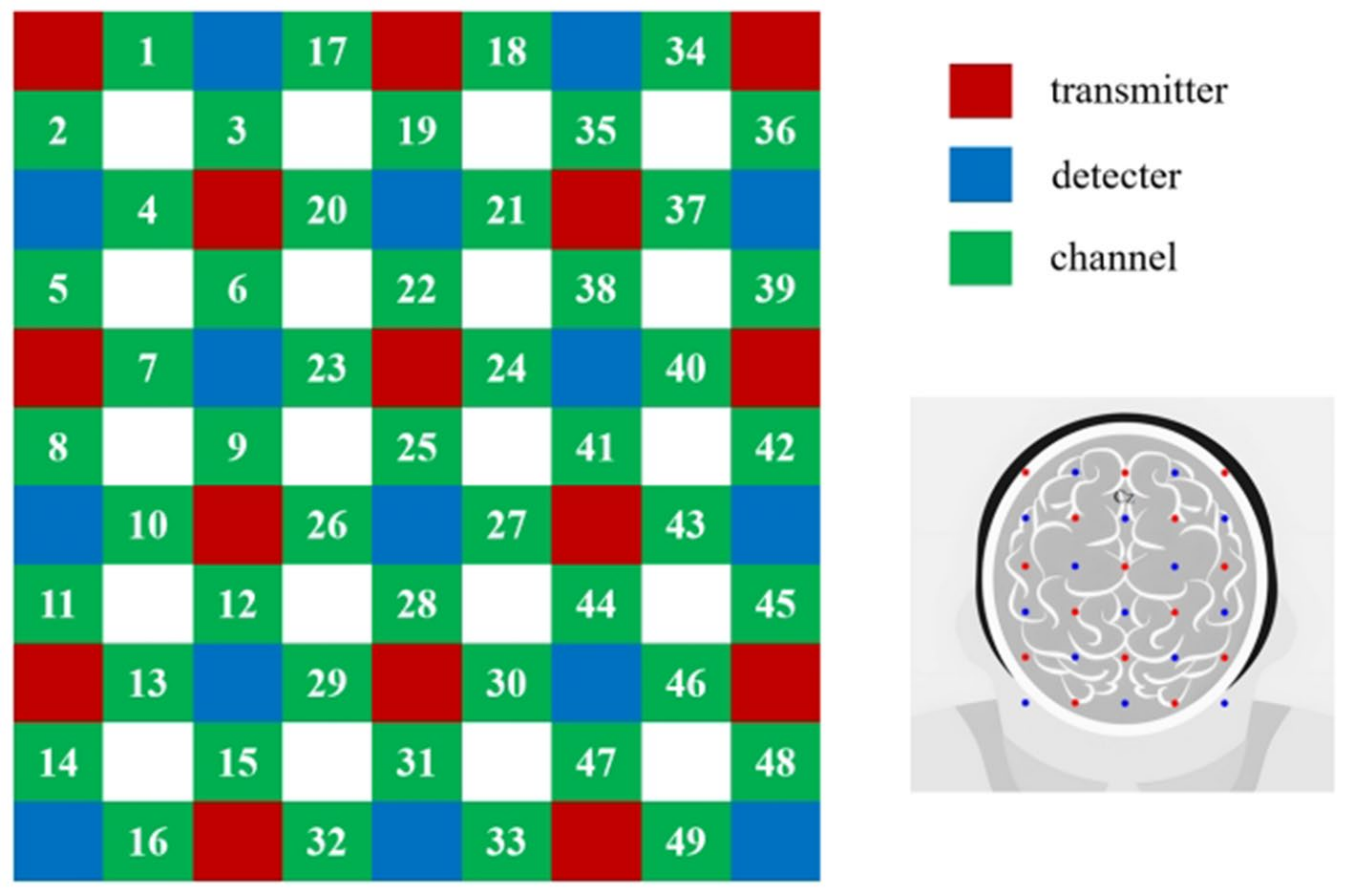
Optode placement. Red indicates the transmitters, blue the receivers, and green the channels.

Using the fNIRS system (SHIMADZU-LABNIRS) equipped with three wavelengths of fNIRS light (780 nm, 805 nm, 830 nm), we collected data at the maximum sampling rate of 27 Hz (Condell et al., 2022) Based on prior research, we identified the frontal, parietal, and occipital lobes as key brain areas involved in stereoscopic visual fatigue. After considering various factors, we chose to focus our data collection on the frontal and parietal lobes (Cai et al., 2017; Richter et al., 2018; Jiakai et al., 2021; Shin et al., 2021; Kang et al., 2022; Wu et al., 2024). To optimize signal quality and spatial coverage, a custom-designed optode helmet was utilized, with 19 channels arranged according to the international 10–20 system. The midline optodes were aligned along the CZ-OZ axis, and channel 19 was positioned at the Cz electrode (vertex). As illustrated in Figure 2, the optode configuration consisted of 12 sources (red), 12 detectors (blue), and 19 channels (green), ensuring comprehensive coverage of the targeted cortical regions.

During experimental sessions, participants executed standardized task paradigms while their hemodynamic responses were continuously recorded via fNIRS. The acquired fNIRS signals, specifically reflecting oxyhemoglobin concentration dynamics, were subjected to preprocessing routines following data acquisition. The modified Beer–Lambert Law (Cope and Delpy, 1988; Ilze and Janis, 2021) was employed to convert changes in optical density (ΔOD) over time (Δt) into changes in oxyhemoglobin (ΔHbO) and deoxyhemoglobin (ΔHbR) concentrations due to the absorption of fNIRS light. The description of Equation 1 is as follows:


(1) $$\begin{array}{l} {}[\begin{array}{c} \text{ΔHbR}\\ \text{ΔHbO} \end{array}]=\frac{1}{d}{\left[\begin{array}{c} \text{εHbR}(\lambda_{1})\text{εHbO}(\lambda_{1})\\ \text{εHbR}(\lambda_{2})\text{εHbO}(\lambda_{2}) \end{array}\right]}^{-1}\\ \left[\begin{array}{c} \mathrm{\Delta OD}(\mathrm{\Delta t},\lambda_{1})/\mathrm{DPF}(\lambda_{1})\\ \mathrm{\Delta OD}(\mathrm{\Delta t},\lambda_{2})/\mathrm{DPF}(\lambda_{2}) \end{array}\right] \end{array}$$

Where d represents the distance between the transmitter and detector, *λ*1 and λ2 represent the different irradiation wavelengths, DPF is the differential path length factor for λ and *ϵ* is the extinction coefficient for HbR and HbO.

Total hemoglobin concentration (∆HbT) was calculated as the sum of the concentrations of oxyhemoglobin (∆HbO) and deoxyhemoglobin (∆HbR). Finally, we applied a digital filtering protocol to eliminate physiological noise and motion artifacts during the fNIRS signal acquisition process.

#### 2.2.3 Experiment protocol

**Figure 3:**
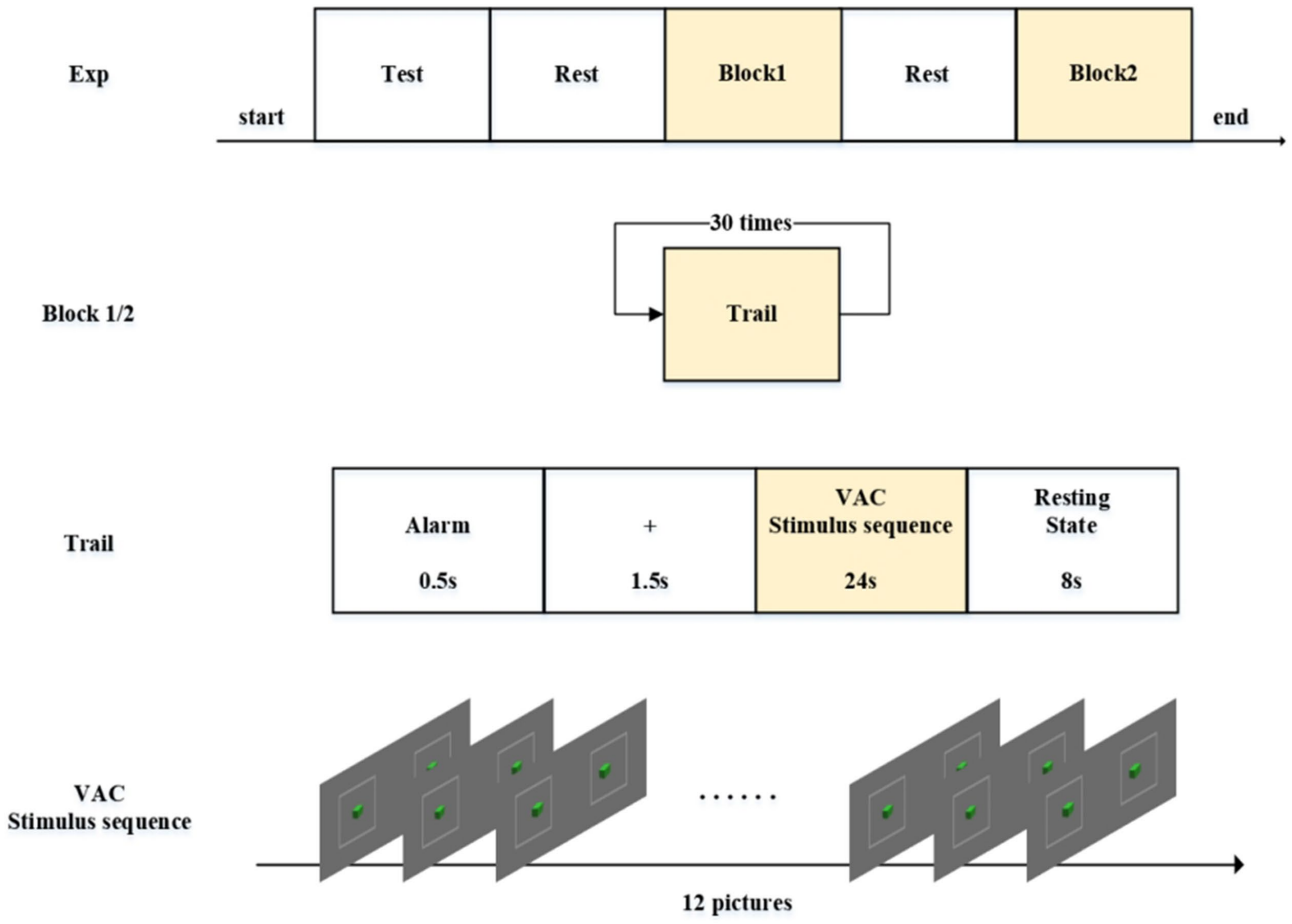
Experiment protocol. The experiment consists of five parts. Both Block 1 and Block 2 contain 30 repeated trials each. Each trial is composed of four segments. The VAC stimulus sequence consists of 12 images with alternating disparity changes.

The experimental protocol comprised five distinct phases as schematically illustrated in Figure 3, programmed and delivered via E-Prime 2.0 (Psychology Software Tools). During the initial verification phase, participants viewed all stereoscopic disparity images presented in a counterbalanced pseudorandom sequence. This design served dual purposes: (1) Confirming participants’ capability to accurately perceive depth perception, and (2) ensuring proper image display functionality. Upon successful verification, the subsequent phase incorporated an eye-closed rest interval (minimum 300 s) to allow stabilization of hemodynamic parameters to pre-experimental baseline levels, as monitored through real-time fNIRS biosignal feedback. The third experimental phase (Block 1 stimulation) comprised a 30-trial sequential paradigm with four standardized stages per trial: (1) Hear the buzzer. The experiment starts; (2) Central fixation cross (0.5° visual angle) displayed for 2 s to stabilize ocular position; (3) Computer-generated pseudorandomized sequence of six stereoscopic disparity images (counterbalanced presentation scheme), each displayed twice (200 ms/image) across 12 presentations (total 24-s duration) using E-Prime’s script-controlled presentation; and (4) a closed-eye rest phase lasting 8 s. Participants would hear a beep at the end of each rest period signaling them to open their eyes for the next trial. After completing Block1, participants entered another rest phase—the fourth part of the experiment. The fifth part began after participants pressed the spacebar, featuring visual stimuli with either small (0.1°–0.5°) or large (2.1°–2.5°) disparity ranges as determined by trial phase requirements. Both Phase III and Phase V maintained identical procedural protocols while systematically varying stimulus disparity parameters, enabling comparative analysis of visual comfort metrics and fatigue progression. This experimental protocol received approval from the Ethics Committee of Changchun University of Science and Technology.

#### 2.2.4 Data preprocessing

**Figure 4:**
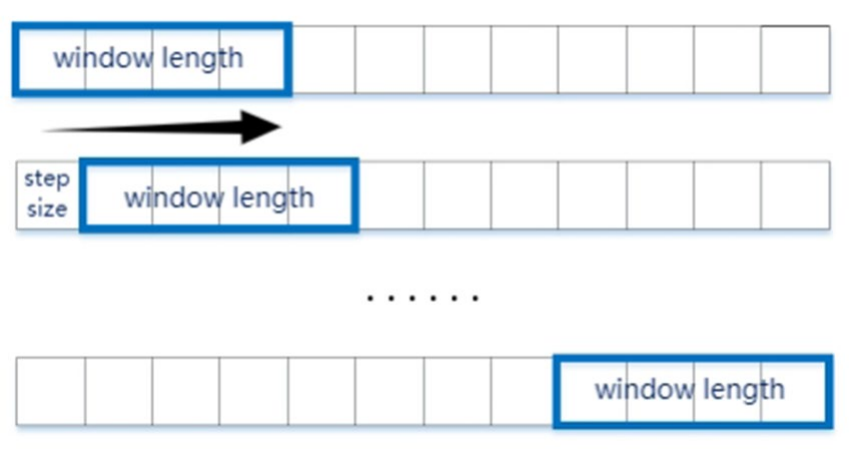
Sliding window method to segment the data samples. The experimental stimuli lasted 8 s, and we selected a window size corresponding to 2 s of data, with a sliding step of 1 s.

To address the temporal demands of fNIRS data acquisition and the extensive dataset requirements of deep learning architectures, we implemented a time-window segmentation strategy for hemodynamic signal processing. Given that fNIRS measurements quantify relative hemodynamic changes [HbO2/HbR], baseline normalization was performed using the initial 10-s resting-state period prior to stimulus onset. Subsequent analysis focused on 24-s epochs corresponding to stereoscopic stimulus presentation, with temporal segmentation executed via an 8-s sliding window (2-s step size; see Figure 4). This method matched our focus on stereoscopic visual fatigue caused by changes in disparity, allowing us to analyze four sets of disparity stimuli changes within the 8-s window, with each step corresponding to the 2-s duration of each stimulus.

### 2.3 Model

#### 2.3.1 DBCNN-ECA-TRM framework

**Figure 5:**
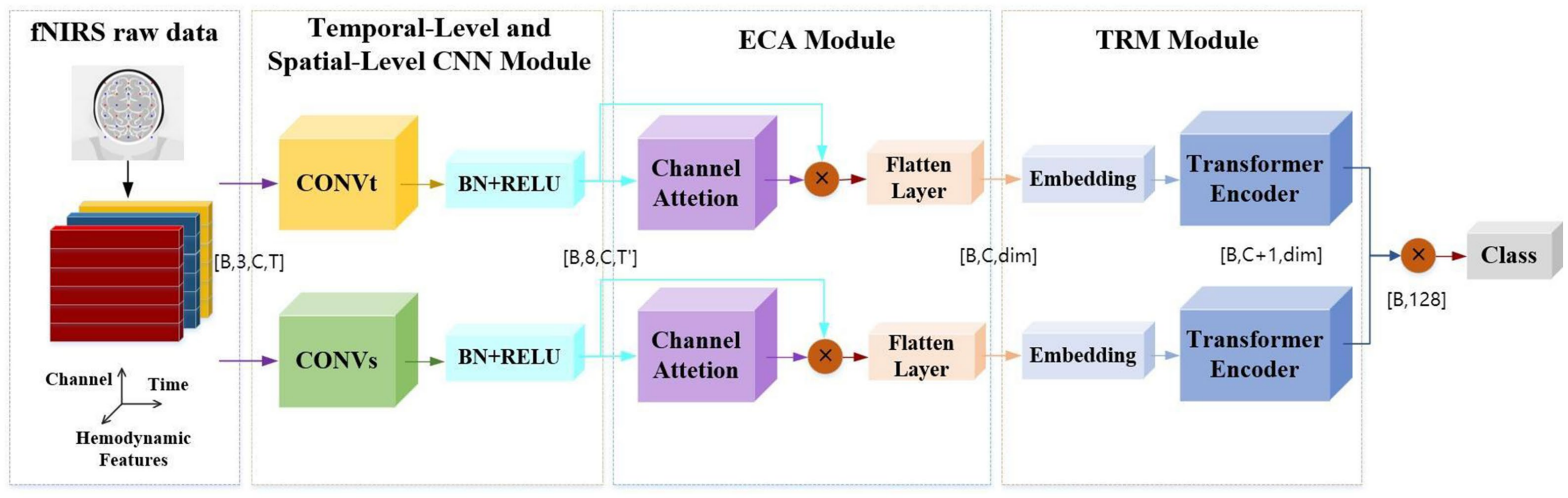
The overall process of DBCNN-ECA-TRM construction.

To effectively extract features from raw fNIRS signals, this study introduces an end-to-end dual-branch convolutional neural network with efficient channel attention and a transformer (DBCNN-ECA-TRM) framework. As schematically depicted in Figure 5, the framework comprises three principal components through which raw fNIRS data undergo hierarchical processing. The input signals are first processed by two specialized CNN modules: A temporal feature extraction module (CONVt) that operates on single-channel time-series data to capture dynamic hemodynamic variations A spatial feature extraction module (CONVs) that analyzes multi-channel spatial patterns across adjacent sensor channels representing localized cortical regions Notably, this dual-branch design enables parallel processing of temporal kinetics and spatial topography inherent in fNIRS signals. The ECA module employs adaptive attention mechanisms to optimize input hemoglobin concentration features (HbO, HbR, and HbT). The TRM module then performs temporal sequence reorganization, converting 2D features into 1D features. This operation preserves critical temporal dependencies while reducing feature dimensionality for efficient processing. The processed 1D feature sequences undergo linear embedding and layer normalization before being fed into the Transformer encoder architecture. Through multi-head self-attention mechanisms and position-wise feedforward networks, this component captures long-range temporal correlations and global signal patterns essential for classification tasks. The design of this model framework effectively captures the key features of stereoscopic visual fatigue in fNIRS signals.

In order to provide a clearer explanation of the model principles, we define symbols here. Assuming that the number of samples input into the model each time is B, the original fNIRS signal is input as a four-dimensional tensor [B, 3, C, T], where C represents the number of channels of the signal and T represents the time sampling point. The model extracts spatiotemporal features in parallel through both spatial and temporal branches. The temporal dimension is compressed to T’, and the output shape is [B, 8, C, T’]. After dynamic weighting through efficient channel attention (ECA), the data shape becomes [B, C, dim], where dim is the defined dimension of the latent space. The original high-dimensional features are compressed to 64 dimensions through linear projection. After passing through the Transformer encoder, the spatial and temporal branches are extended to [B, C + 1, dim] by adding CLS Token, and the dual branches are concatenated to [B, 128]. The final output is [B, n], where n is the number of task categories.

#### 2.3.2 DBCNN module

**Figure 6:**
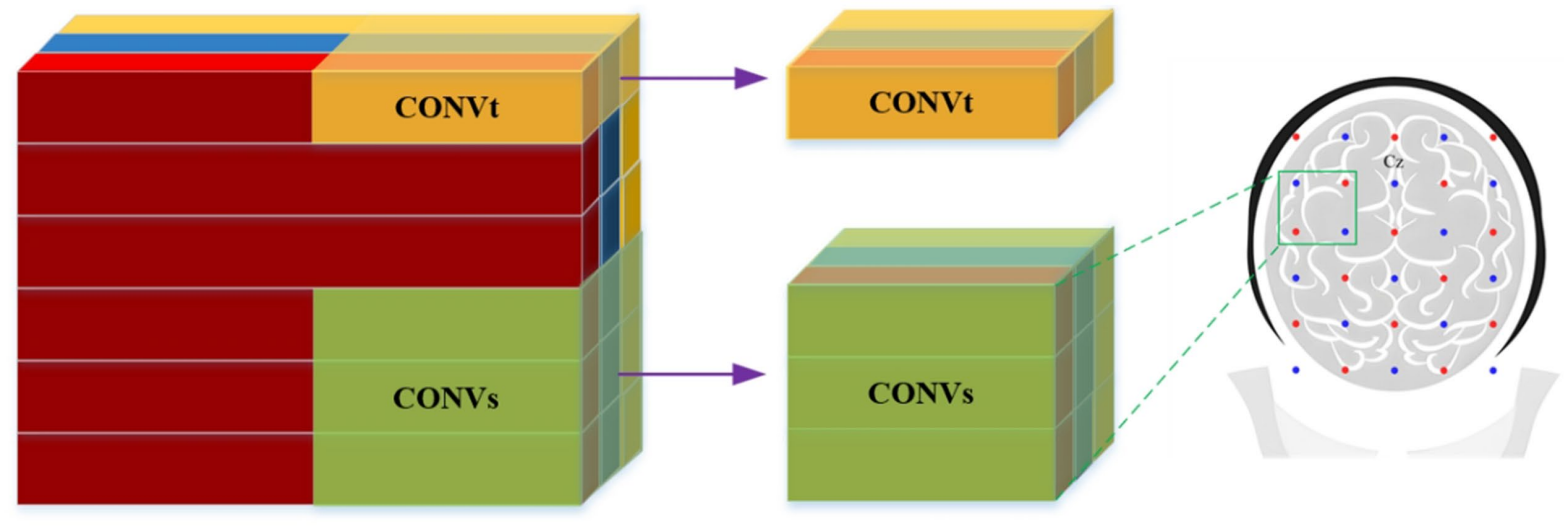
The relationship between temporal and spatial convolutions. Time branch (CONVt): extracting dynamic time features within a single channel; spatial branches (CONVs): extracting spatial correlations between multiple channels; sliding operation of convolution kernel: capture spatiotemporal features by sliding the convolution kernel on the sensor array.

Effectively utilizing fNIRS signals is crucial for improving classification outcomes. Previous studies have demonstrated that optimizing the raw input data of fNIRS signals can enhance the exploration and learning of signal features (Song et al., 2023; Zhang et al., 2023). The core idea of CNN is to extract different features using convolution operations. While conventional CNN architectures employ fixed kernel sizes for localized feature detection, our research introduces a novel dual-branch CNN framework designed to synergistically capture both temporal and spatial characteristics inherent in fNIRS data. As shown in Figure 6, we represent the input fNIRS data as a three-dimensional matrix, indicating channels, time points, and cerebral oxygenation levels (HbO, HbR, and HbT). At the temporal level, we use the CONVt to compute the hemodynamic response of individual channels, focusing on data variations within a single channel. At the spatial level, the CONVs is used to extract information across multiple channels, targeting different brain regions. Through systematic sliding of convolution kernels over the sensor array, this module captures spatially correlated hemodynamic patterns among neighboring channels.The parameter settings for both types of convolutions are detailed in Table 1. The proposed dual-modal extraction strategy not only improves data utilization efficiency through joint temporal–spatial feature integration but also significantly strengthens the network’s representational capacity. This hybrid architecture effectively addresses the limitations of single-method approaches by synergistically preserving local channel-specific patterns while capturing global spatial correlations inherent in fNIRS data. Such complementary feature learning enables the network to achieve superior classification performance through more robust representation of stereoscopic visual fatigue signatures.

**Table 1:** Parameter settings of the convolutions.

Convolution	Size	Filter	Stride
CONVs	(3, 30)	8	(3, 3)
CONVt	(1, 30)	8	(1, 3)

#### 2.3.3 ECA module

The Efficient Channel Attention (ECA) mechanism was developed to address the challenges of insufficient utilization and imbalanced attention allocation across feature channels in deep neural networks, particularly in image processing applications (Wang et al., 2020). Traditional networks often assign equal weight to all channels, resulting in suboptimal feature extraction and reduced model efficiency. The ECA mechanism overcomes this limitation by enabling the network to dynamically allocate attention weights to individual channels, thereby enhancing the focus on critical features and improving both performance and generalization capabilities. In fNIRS signal analysis, where channels such as HbO, HbR, and HbT reflect hemoglobin oxygenation during brain activity. By adaptively weighting these channels, ECA facilitates the precise extraction of physiologically relevant features, thereby enhancing the accuracy and reliability of brain function analysis.

**Figure 7:**
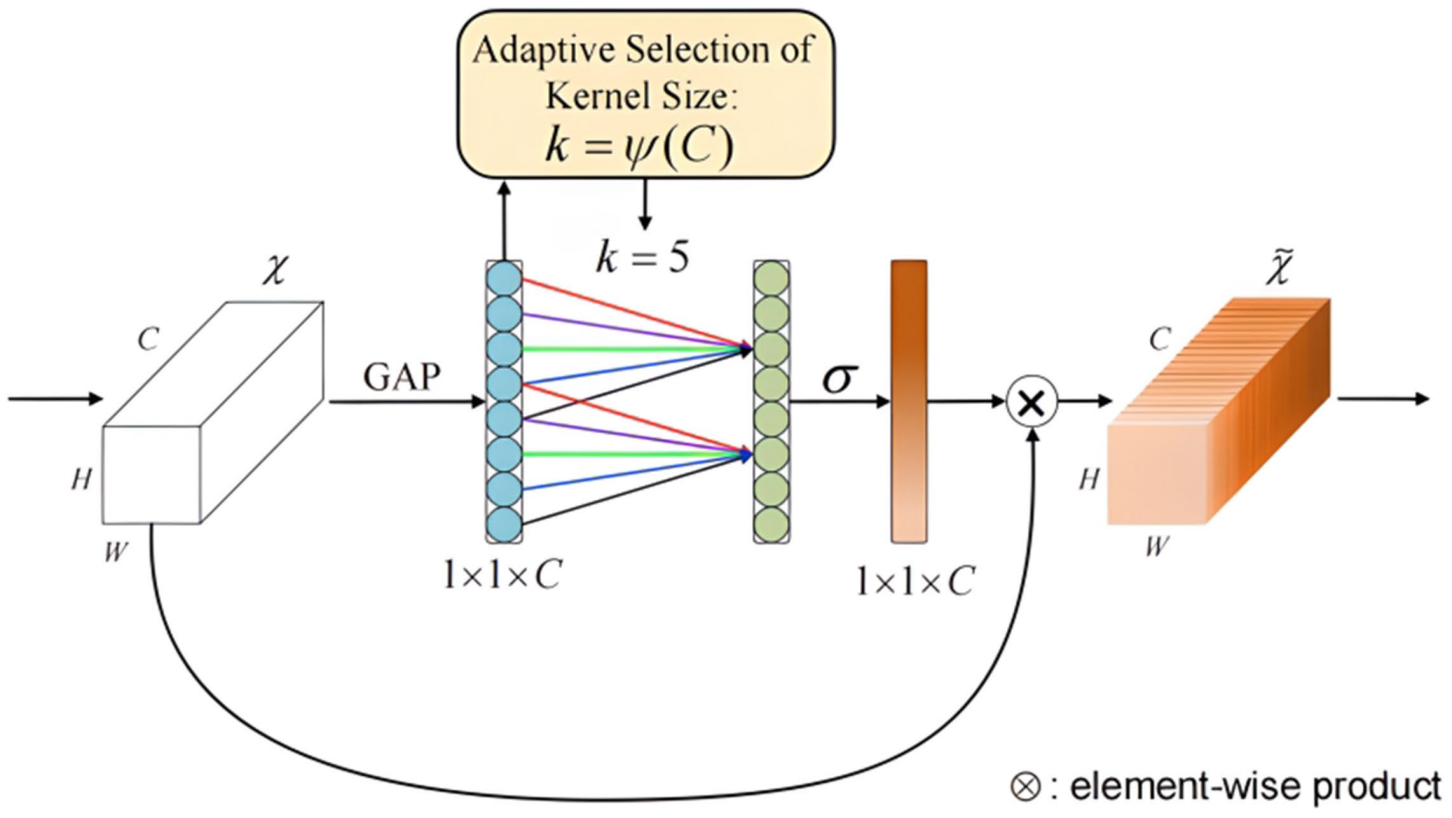
Structure of the efficient channel attention module. Structure of efficient channel attention module. The ECA module includes global average pooling, 1D convolution, and sigmoid activation. Enhance the extraction of key features through dynamic weighted channels.

Figure 7 depicts the architectural design of the Efficient Channel Attention (ECA) module, utilizing 1D convolution to efficiently achieve local cross-channel interaction based on the dependency relationships between fNIRS signal feature channels. As shown, the ECA attention module first performs global average pooling on the input data of dimensions H × W × C, followed by a 1D convolution operation using a convolution kernel of size k. The kernel size k is determined by an adaptive function of the number of input channels C, as shown in Equation 2, where |x|_odd_ represents the nearest odd number to x.


(2) $$k=\varphi (c)={|\frac{\log_{2}c+1}{2}|}_{\mathrm{odd}}$$

Following the convolution operation, a sigmoid activation function is applied to obtain the weights W for each channel. To further enhance network performance, convolutional weights are shared to efficiently capture local inter-channel interactions, reducing the number of network parameters. The shared weights method is detailed in Equation 3,


(3) $$W_{i}=\sigma (\sum_{j=1}^{k}W_{i}^{j}Y_{i}^{j}),Y_{i}^{j}\in \Omega_{i}^{k}$$

where *σ* represents the sigmoid activation operation, W_i_ is the i-th weight matrix obtained by grouping the C channels, W_i_^j^ is the j-th local weight matrix within the i-th weight matrix, and Y_i_^j^ is defined similarly. Finally, the obtained weights are multiplied by the original input feature map to produce a feature map with attention weights. As a plug-and-play module, the ECA attention mechanism employs a straightforward concept and computation, minimizing the impact on network processing speed while significantly improving classification accuracy.

Incorporating the ECA module allows the model to dynamically assess the importance of each feature channel, improving its focus on crucial channels. This integration enhances the model’s ability to represent fNIRS data and significantly improves the accuracy and reliability of brain function analysis.

#### 2.3.4 TRM module

**Figure 8:**
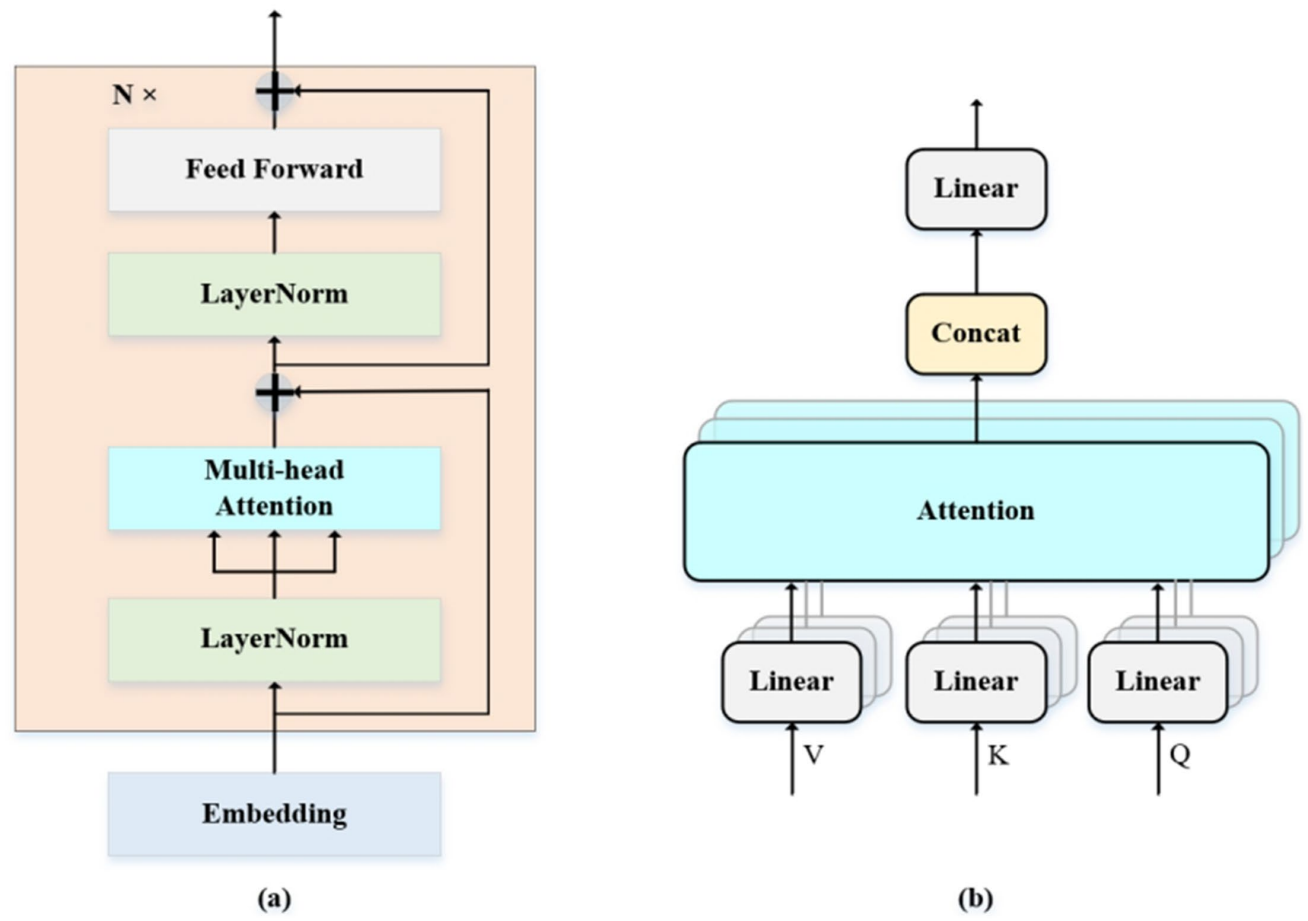
**(a,b)** Are the structure of Transformer encoder and MSA. **(a)** The overall architecture of Transformer encoder includes two core sub layers: multi-head self-attention (MSA) and multi-layer perceptron (MLP). **(b)** The detailed process of the self-attention calculation module includes steps such as linear projection, attention score calculation, Softmax normalization, and weighted summation.

Figure 8a schematically depicts the architecture of the Transformer encoder, which comprises an array of identical encoding layers. Each containing two sub-layers: multi-head self-attention (MSA) and a multi-layer perceptron (MLP). Figure 8b provides a detailed illustration of the self-attention computation module, where the input tensor undergoes linear projection to generate attention queries Q, keys K, and values V. Self-Attention is defined as Equation 4:


(4) $$\text{Attention}(Q,K,V)=\text{softmax}(\frac{{\mathrm{QK}}^{T}}{\sqrt{d_{k}}})V$$

Where d_k_ is the dimension of k. The MSA consists of h parallel self-attention layers. Defined as Equations 5, 6:


(5) $$\mathrm{MSA}(Q,K,V)=\text{Concat}({\text{head}}_{1},\ldots ,{\text{head}}_{h})W^{O}$$

(6) $${\text{head}}_{i}=\text{Attention}(QW_{i}^{Q},KW_{i}^{K},VW_{i}^{V})$$

Where W_i_^Q^, W_i_^K^, W_i_^V^ and W^O^ are parameter matrices. The MLP contains two linear layers with Gaussian Error Linear Unit (GELU; Mei et al. 2022) activation functions. It is defined as Equation 7:


(7) $$\mathrm{MLP}(x)=\text{GELU}({\mathrm{xW}}_{1}+b_{1})W_{2}+b_{2}$$

The model architecture consists of 6 identical layers, each containing two fully connected layers (W_1_ and W_2_) with a hidden dimension of 64, and bias terms (b_1_ and b_2_). Layer normalization is critically applied before both the 8-headed multi-head self-attention (MSA) and the multi-layer perceptron (MLP) sub-layers (with an MLP dimension of 64) to ensure training stability and accelerate convergence, a modification that has proven more effective for training transformers. To further enhance information propagation and mitigate the vanishing gradient problem inherent in deep architectures, residual connections with learnable scaling factors are integrated into each sub-layer (Castaldi et al., 2020).

## 3 Result

### 3.1 Model training and model evaluation

#### 3.1.1 Model training

Our model was extensively trained over 120 epochs with a batch size of 128. For optimization, we selected the Adam optimizer (Richter et al., 2018; Huang et al., 2022; Mei et al., 2022), configured with an initial learning rate of 0.001, decay parameters β1 = 0.9 and β2 = 0.999, and a weight decay of 0.01. To enhance generalization and reduce overfitting, label smoothing regularization was integrated into the training pipeline.

#### 3.1.2 Model evaluation

To assess the model’s classification performance, we utilized two commonly recognized metrics: accuracy and kappa. Accuracy quantifies the fraction of correctly classified samples relative to the total dataset size, offering a fundamental assessment of general classification performance. Cohen’s kappa (*κ*) provides a more rigorous evaluation by comparing the observed classification accuracy against the expected accuracy derived from random chance, thereby controlling for statistical agreement occurring by chance. This metric offers a more robust measure of model performance, particularly useful in datasets with imbalanced class distributions. The definitions of these indicators are shown in Equations 8 and 9:


(8) $$\text{accuracy}=\frac{\mathrm{TP}+\mathrm{TN}}{\mathrm{TP}+\mathrm{FN}+\mathrm{FP}+\mathrm{TN}}$$

(9) $$\text{Kappa}=\frac{P_{0}-P_{e}}{1-P_{e}}$$

TP (true positive) refers to the instances where the original data is classified as positive and remains positive after classification. TN (true negative) denotes the instances where the original data is classified as negative and remains negative after classification. FN (false negative) represents the instances where the original data is classified as positive but is classified as negative after classification. FP (false positive) indicates the instances where the original data is classified as negative but is classified as positive after classification. In terms of kappa value, within the confusion matrix, $P_{0}=\frac{\sum_{i=1}^{n}a_{\mathrm{ii}}}{N}$ , $P_{e}=\frac{\sum_{i=1}^{n}a_{i+}*a_{+i}}{N^{2}}$ . Among them $a_{i+}=\sum_{j}\mathrm{aij}$ , $\;\;a_{+j}=\sum_{j}a_{\mathrm{ij}}$ , and n is the number of categories, N is the total number of samples.

### 3.2 Classification result

**Figure 9:**
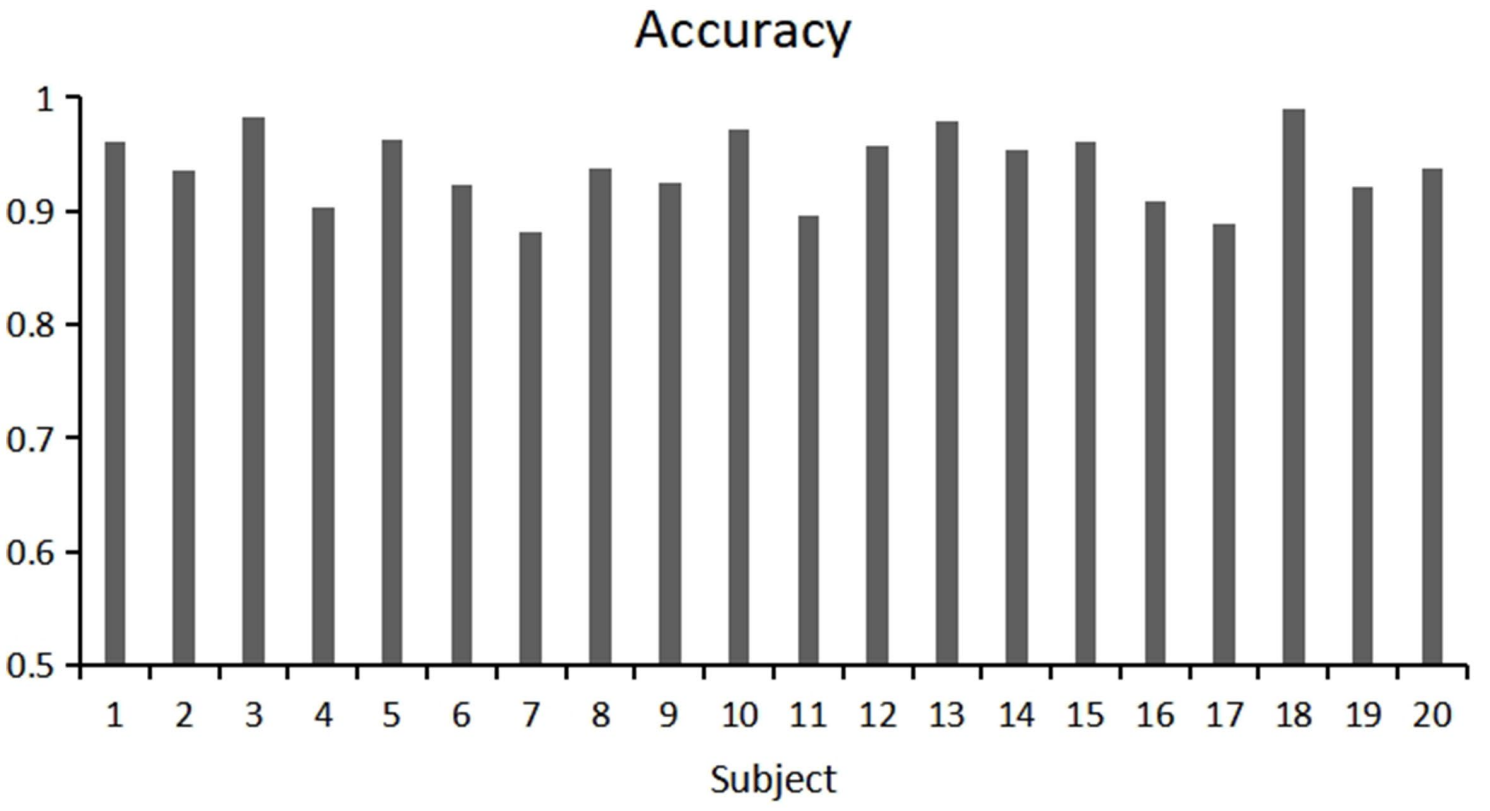
Classification accuracy of each participant under 5-fold cross validation.

**Figure 10:**
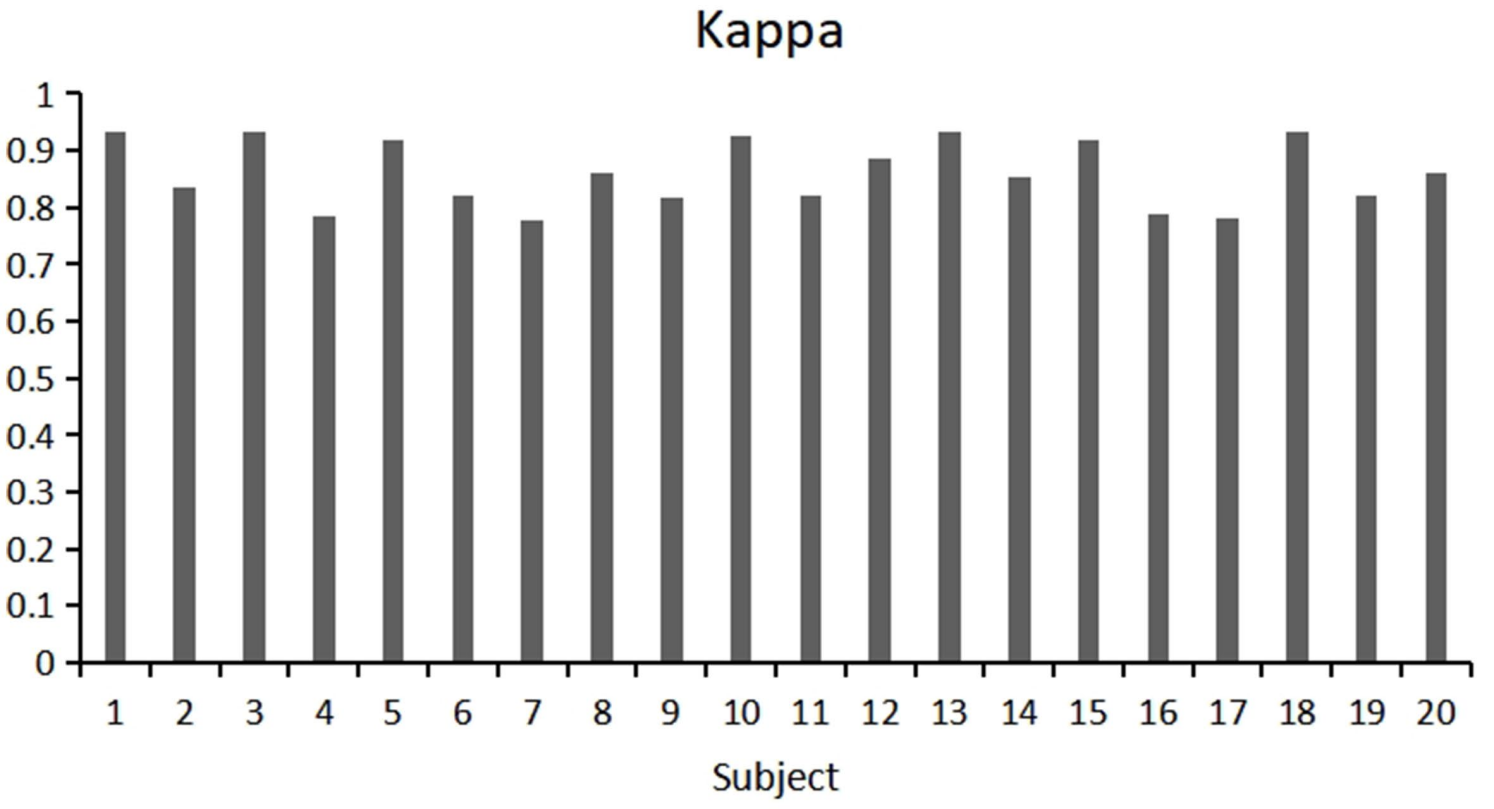
Kappa values of each participant under 5-fold cross validation.

This study used a fivefold cross validation method, repeated five times, to evaluate the classification accuracy and kappa value of each participant. Show the variability of participants’ responses to stereoscopic visual stimuli. As shown in Figures 9, 10. Notably, Participant 18 demonstrated the highest classification accuracy at 98.96%, while Participant 7 had the lowest accuracy at 88.23%. Overall, the classification performance among participants was robust, with an overall average accuracy of 93.12% and an average kappa value of 0.83. These outcomes highlight the robustness and stability of our model, showcasing consistently good classification performance across different participants.

### 3.3 Ablation study

To validate the rationality of the proposed model, we conducted an ablation study involving configurations with only temporal convolution (CONVt), only spatial convolution (CONVS), no effective channel attention (no ECA) module, and no transformer (no TRM) module. As shown in Table 2, after training and evaluating these configurations, we found that deleting different modules had varying degrees of impact on model performance. Especially, removing the transformer module resulted in a significant decrease in accuracy from 93.12 to 84.65%, indicating that transformers play a crucial role in capturing long-distance temporal dependencies. Although Transformer has advantages in long sequence modeling, its application premise is that the data itself has long-range dependencies. This article confirms this premise through spectral analysis (*β* = 0.9), while ablation experiments show that adding the Transformer model improves performance (+8.47%). This result not only confirms the long-term dependence of fatigue signals, but also explains why dismantling transformers leads to a significant decrease in performance. In addition, the ECA module dynamically suppresses redundant features (such as high-frequency noise) through channel attention weights, focusing on the blood oxygen response at critical time steps. After removing ECA (NO-ECA), the model was unable to distinguish between informative and noisy channels, resulting in a decrease in classification accuracy to 89.91%, indicating the importance of dynamic channel weighting for classification performance. Specifically, through the analysis of channel weights, the ECA module significantly increased the weight of the HbO channel (from 0.35 to 0.62), while reducing the weights of HbR and HbT (from 0.33 and 0.32 to 0.21 and 0.17, respectively). The increase in HbO weight by the ECA module may be related to the accumulation of oxygenated hemoglobin caused by increased metabolic demand in brain regions during the late stage of fatigue. Although combining all feature signals is usually better than selecting only one type, it may introduce information redundancy. For example, the joint input of HbO, HbR, and HbT may result in high correlation between features. The application of ECA channel attention mechanism to fNIRS feature signals effectively alleviates this problem and improves classification accuracy.

**Table 2:** Comparison results of ablation experiments.

Model	ACC	Kappa
Only-CONVt	89.58	0.79
Only-CONV_S_	85.41	0.70
No-ECA	89.91	0.76
No-TRM	84.65	0.71
DBCNN-ECA-TRM	93.12	0.83

### 3.4 Comparative experiment

Currently, the research and application of fNIRS technology in the field of deep learning are not yet systematic, with a noticeable absence of comparable models. No studies have applied deep learning to research stereoscopic visual fatigue using fNIRS technology or validated the proposed models’ performance advantages. In this study, we used traditional classifiers for comparison, including k-nearest neighbors (KNN) with k set to 50, an artificial neural network (ANN) with 128 hidden layer units capped at 10,000 iterations, and a support vector machine (SVM) employing a radial basis function kernel with a regularization parameter of 1. These baseline machine-learning models were implemented using the Scikit-Learn package.

Additionally, we evaluated classic deep learning models such as Convolutional Neural Networks (CNN) with three 1D convolutional layers (kernel sizes of 3/5/7), two fully connected layers (64 nodes each), and a softmax layer; Long Short-Term Memory Recurrent Neural Networks (LSTM) with 128 hidden layers; and a Transformer-Encoder network with a multi-head self-attention matrix and two fully connected layers (64 nodes each). All pre-trained deep learning models were sourced from the PyTorch image models (Timm) package, obtained from an online open-source repository.

As shown in Table 3, comparative results demonstrated a significant advantage of deep learning models over traditional machine learning models. Although SVM performed well within the machine learning category, its accuracy was at least 10% lower than that of the deep learning models, and its kappa value was also comparatively lower. CNN performed the best among the deep learning models. This result is likely due to its proficiency in extracting local features in short-term visual stimulus tasks using our time window processing approach. Transformer and LSTM, typically stronger at capturing features from long-distance signal changes, did not show significant advantages in this context. However, the Transformer performed second best, possibly due to its self-attention mechanism allowing it to better focus on key points in the input sequence. Our proposed model combines attention mechanisms with local feature extraction and demonstrates superior performance, confirming its suitability for assessing stereoscopic visual fatigue.

**Table 3:** Comparison results with other models.

Model	ACC	Kappa
KNN	59.17	0.19
ANN	65.83	0.33
SVM	68.33	0.42
CNN	83.25	0.65
LSTM	77.50	0.54
Transformer	82.08	0.62
DBCNN-ECA-TRM	93.12	0.83

### 3.5 Cross-subject classification result

This study used cross-subject measurement to assess the model’s accuracy. This approach minimizes individual differences and enhances the robustness of the model assessments. Specifically, we implemented two cross-subject evaluation strategies: 5-fold cross-validation and leave-one-subject-out (LOSO) cross-validation. These strategies provided a more accurate assessment of the model’s generalization performance and classification ability.

The classification results, as detailed in Table 4, demonstrate that the model performs well in cross-subject accuracy assessments. In the 5-fold cross-validation, our model achieved an accuracy of 87.42%, while in the LOSO evaluation, it reached an accuracy of 83.91%. These outcomes indicate that the model possesses strong generalization capabilities across different subjects and can perform effectively in real-world applications. This robust performance provides reliable support for our study and further confirms the effectiveness and reliability of the proposed model.

**Table 4:** Average classification results across subjects.

Training strategy	Acc
5-FOLD	87.42
LOSO	83.91

**Figure 11:**
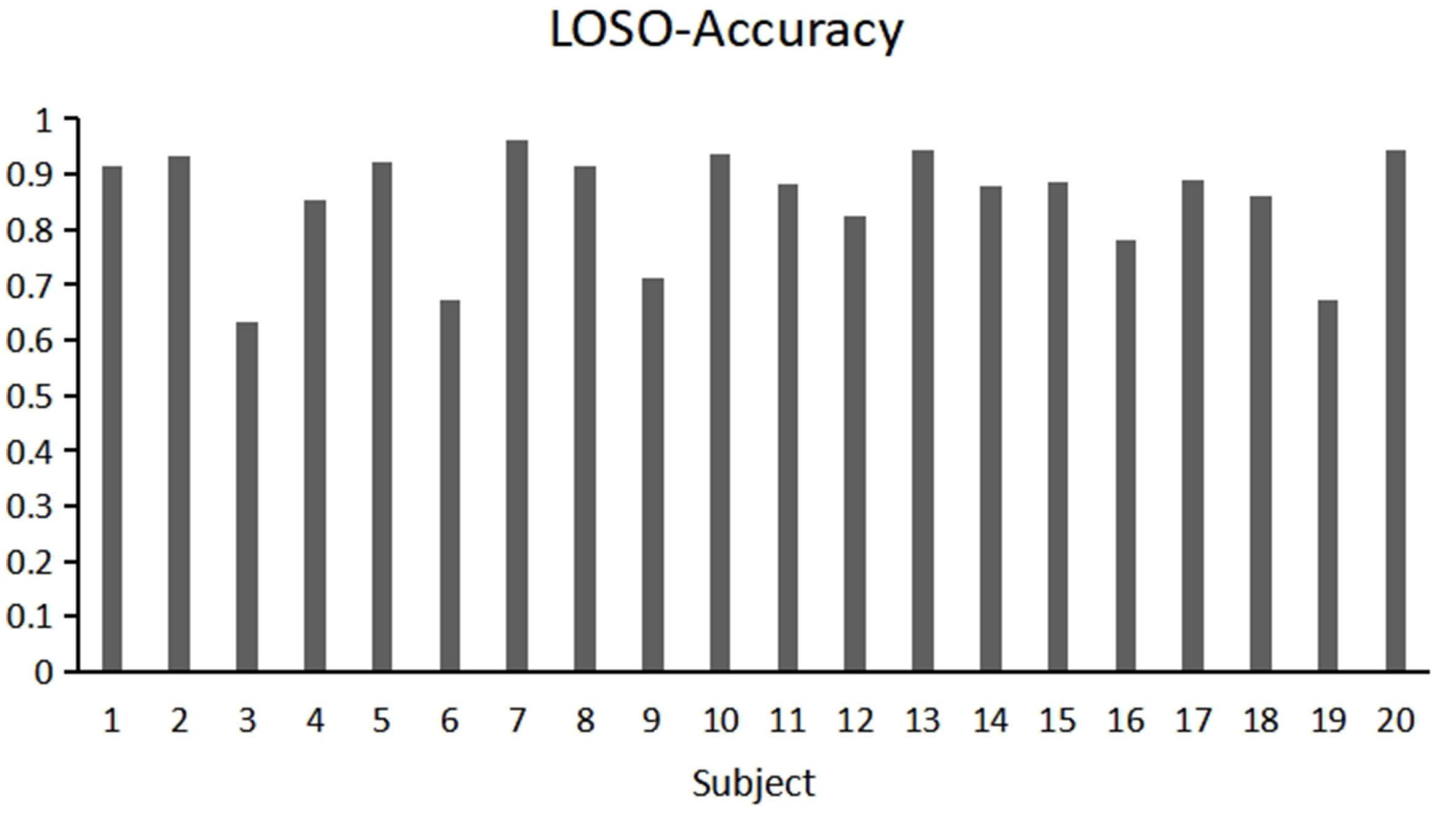
Individual classification results of 20 participants using the Leave-One-Subject-Out (LOSO) method.

Moreover, we observed significant fluctuations in accuracy among different subjects when employing the LOSO (leave-one-subject-out) validation for cross-subject verification. Specifically, Figure 11 shows the accuracy of different subjects in cross subject validation, the highest accuracy recorded was 96.25% (Subject 7), while the lowest was only 63.33% (Subject 3). We assume that different brain regions of subjects have different responses to the same stereoscopic stimulus (e.g., delayed or insufficient activation of the occipital visual cortex in some individuals), leading to significant differences in classification results. This inter individual heterogeneity may stem from atypical hemodynamic response patterns, such as delayed HbO peak or abnormal HbR signal fluctuations, which may reflect individual specificity of neurovascular coupling efficiency or anatomical structures. This variability suggests that compared to 5-fold cross-validation, using LOSO validation for cross-subject verification may result in greater fluctuations in accuracy. This finding highlights the need to consider individual differences in neuroimaging studies, especially when validating models for broad application.

## 4 Conclusion

fNIRS is a non-invasive neuroimaging technology with higher spatial and temporal resolution and less noise interference than EEG and fMRI. This paper introduces a stereoscopic visual fatigue stimulation paradigm and employs a deep neural network model for end-to-end feature extraction and classification. We improved classification accuracy by at least 10% compared to traditional machine learning approaches. The proposed network model outperformed conventional deep learning models, offering significant advancements in the field. This study is the first to apply deep learning to assess stereoscopic visual fatigue using fNIRS, addressing a key research gap.

## Data Availability

The raw data supporting the conclusions of this article will be made available by the authors, without undue reservation.
